# Association between skin diseases and severe bacterial infections in children: case-control study

**DOI:** 10.1186/1471-2296-7-52

**Published:** 2006-08-31

**Authors:** Robbert SA Mohammedamin, Johannes C van der Wouden, Sander Koning, Sten P Willemsen, Roos MD Bernsen, François G Schellevis, Lisette WA van Suijlekom-Smit, Bart W Koes

**Affiliations:** 1 Department of General Practice, Room FF 304, Erasmus MC-University Medical Center Rotterdam, PO Box 1738, 3000 DR Rotterdam, The Netherlands; 2 Department of General Practice, Free University Amsterdam/NIVEL, Netherlands Institute for Health Services Research Utrecht, PO Box 1568, 3500 BN Utrecht, The Netherlands; 3 Department of Paediatrics, Erasmus MC-University Medical Center/Sophia Children's Hospital. Rotterdam, PO Box 2040, 3000 CA Rotterdam, The Netherlands

## Abstract

**Background:**

Sepsis or bacteraemia, however rare, is a significant cause of high mortality and serious complications in children. In previous studies skin disease or skin infections were reported as risk factor. We hypothesize that children with sepsis or bacteraemia more often presented with skin diseases to the general practitioner (GP) than other children. If our hypothesis is true the GP could reduce the risk of sepsis or bacteraemia by managing skin diseases appropriately.

**Methods:**

We performed a case-control study using data of children aged 0–17 years of the second Dutch national survey of general practice (2001) and the National Medical Registration of all hospital admissions in the Netherlands. Cases were defined as children who were hospitalized for sepsis or bacteraemia. We selected two control groups by matching each case with six controls. The first control group was randomly selected from the GP patient lists irrespective of hospital admission and GP consultation. The second control group was randomly sampled from those children who were hospitalized for other reasons than sepsis or bacteraemia. We calculated odds ratios and 95% confidence intervals (CI). A two-sided p-value less than 0.05 was considered significant in all tests.

**Results:**

We found odds ratios for skin related GP consultations of 3.4 (95% CI: [1.1–10.8], p = 0.03) in cases versus GP controls and 1.4 (95% CI: [0.5–3.9], p = 0.44) in cases versus hospital controls. Children younger than three months had an odds ratio (cases/GP controls) of 9.2 (95% CI: [0.81–106.1], p = 0.07) and 4.0 (95% CI: [0.67–23.9], p = 0.12) among cases versus hospital controls. Although cases consulted the GP more often with skin diseases than their controls, the probability of a GP consultation for skin disease was only 5% among cases.

**Conclusion:**

There is evidence that children who were admitted due to sepsis or bacteraemia consulted the GP more often for skin diseases than other children, but the differences are not clinically relevant indicating that there is little opportunity for GPs to reduce the risk of sepsis and/or bacteraemia considerably by managing skin diseases appropriately.

## Background

Sepsis or bacteraemia requiring hospital admission is rare, however it is a significant cause of high mortality and serious complications such as septic shock and multi organ dysfunction syndrome [1-3]. Currently, little data is available about the causal factors of sepsis or bacteraemia in children in the population. The available studies in this field deal particularly with adults or with children belonging to high-risk groups such as neonates and those who are immunocompromized due to HIV infection and children with underlying malignancies [4-7]. The few studies which have been performed on sepsis or bacteraemia in children from the general population are case series [8-10] or deal with specific causative bacterial agents [1,11-13].

Three previous studies of which only one performed in children reported that from the identifiable primary focus in patients with sepsis or bacteraemia most often (22–37%) an infection of the skin was detected [1,2,12]. Children suffering from atopic dermatitis are chronic carriers of *Staphylococcus Aureus *and run therefore a higher risk to develop sepsis or bacteraemia [9,14]. Skin infections are almost always curable, but some may lead to serious complications such as nephritis, carditis, arthritis and sepsis if the diagnosis is delayed and/or treatment is inadequate [15].

A Dutch study performed in children aged 0–14 years reported that 28% of those with skin diseases consulted the general practitioner (GP) [16]. Hence, for this reason, we hypothesize that children who were admitted to hospital due to sepsis or bacteraemia suffered more often from skin diseases, especially skin infections, and therefore visited their GP for this reason more often prior to their admission compared to their controls. If our hypothesis is true and given the fact that skin diseases account for 23% of the total morbidity in children in general practice [17], the GP may be able to reduce the risk of sepsis or bacteraemia by recognizing skin diseases in time and treating them adequately.

To test this hypothesis we performed a case-control study, aiming to answer the following research question:

- Did children who were admitted to a hospital for sepsis or bacteraemia visit their GP more often for skin diseases before their admission, compared to matched controls?

## Methods

We used data of the second Dutch National Survey of general practice performed by NIVEL (Netherlands Institute for Health Services Research) in 2001 and data of the LMR (National Medical Registration in the Netherlands).

### Second Dutch National Survey

In the Netherlands, general practices have a fixed list size and all inhabitants are listed with a general practice, and GPs have a gate-keeping role. Usually, the first contact with health care, in a broad sense, is the contact with the general practitioner. This survey included a representative sample of the Dutch population. Data about all physician-patient contacts, prescriptions and referrals during 12 months in 2001 were extracted from electronic medical records of all listed patients of 104 practices (195 GPs) [18]. All diagnoses were coded using the International Classification of Primary Care (ICPC) [19]. Different health problems within one consultation were recorded separately. Socio-demographic characteristics such as age, gender, region and urbanization level of all patients listed to the participating GPs were derived from the GP's computerized patient file. The degree of urbanization was derived from the general practice's postal code and categorized into four classes 'under 30,000 inhabitants', '30,000–50,000 inhabitants', 'over 50,000 inhabitants' and 'the three large Dutch cities Amsterdam, Rotterdam and The Hague'. The Netherlands were divided into a Northern, Central and Southern region. Childrens' socioeconomic status (SES) and ethnic origin were obtained by a questionnaire filled out by parents or by the children themselves if they were older than 12 years (response rate 76%). SES was based on the father's occupation, which was categorized into five classes "non-manual work high (class I)", "non-manual work middle (class II)", "non-manual low and farmers (class III)", "manual work high/middle (class IV)" and "manual work low (class V)". Ethnicity was derived from the country of birth of either parent. If either parent was born in Turkey, Africa, Asia (except Japan and Indonesia) and Central or South America, their children were considered to be children of non-Western origin (in accordance with the classification of Statistics Netherlands). All other children were defined as Western. Eight practices were excluded from analysis because of insufficient quality of data registration.

### LMR (National Medical Registration in the Netherlands)

This continuous registration contains information about hospital admissions, diagnostic and therapeutic interventions of all hospitals in the Netherlands. All diagnoses were coded using the International Classification of Diseases 9^th ^revision (ICD-9) [20]. Previous research revealed that about 87% of the patients referred by the GP to a specialist can be linked to a record of the hospital register [21].

### Cases and controls

Cases were defined as being diagnosed with sepsis or bacteraemia at discharge. The corresponding ICD-9 codes for sepsis and bacteraemia are listed in a separate table [see Additional file 1]. Cases were only selected when their admission date was at least 14 days after the start and before the end of the one-year registration period of the survey in general practice. If cases had more than one admission within a week concerning the same health problem only the first admission was selected. We excluded all children who were primarily admitted to a hospital for skin diseases (N = 29), but assessed GP consultations of these children 14 days prior to their hospital admission.

We selected two control groups by matching each case with six controls. Cases and controls were matched on age group (table 1), gender and region. The first control group was randomly selected from the GP patient lists irrespective of hospital admission and GP consultation, the so called GP controls. The second control group was composed by drawing a random sample from those children who were admitted to a hospital for other reasons than sepsis or bacteraemia, the so called hospital controls. This second control group was added because we can not rule out that some of our severely ill cases bypassed the general practitioner prior to their hospital admission which might lead to an under-estimation of contacts with the GP in this group.

**Table 1:** Baseline characteristics in percentages of cases and controls

	Cases (N = 101)	GP Controls^1 ^(N = 597)	Hospital Controls^2 ^(N = 583)
**Age group**			
0 – 3 months	8.9	7.7	9.3
3 – 6 months	6.9	6.9	5.8
6 – 24 months	27.7	30.2	28.3
24 – 72 months	27.7	26.8	26.8
6 – 17 years	28.7	28.5	29.8
**Gender**			
Boys	63.4	63.7	64.3
Girls	36.6	36.3	35.7
**Urbanization**			
< 30,000	36.6	38.0	36.4
30,000 – 50,000	18.8	15.9	17.5
> 50,000	37.6	39.2	36.9
Big cities^3^	6.9	6.9	9.3
**Region**			
Northern	19.8	20.1	18.0
Central	61.4	60.8	62.4
Southern	18.8	19.1	19.6
**SES**^**4**^			
Non-manual high	34.1	37.4	38.8
Non-manual middle	31.8	31.3	35.6
Non-manual low & farmers	15.9	13.5	5.0
Manual high/middle	2.3	7.5	9.6
Manual low	15.9	10.3	11.0
**Ethnicity**			
Natives & Western immigrants	85.7	89.8	87.2
Non – Western immigrants	14.3	10.2	12.8

1 = control group randomly sampled from the general practitioners' (GP) patient lists irrespective of hospital admission and GP consultation

2 = control group randomly sampled from those children who were hospitalized for other reasons than sepsis or bacteraemia

3 = Amsterdam, Rotterdam, The Hague

4 = according to fathers occupation

### Ethical approval

The study was carried out according to Dutch legislation on privacy. The privacy regulation of the study was approved by the Dutch Data Protection Authority. According to Dutch legislation, obtaining informed consent is not obligatory for observational studies.

### Data-analysis

We analyzed data of all children aged 0–17 years and assessed whether a higher proportion of cases visited the GP with any disease, especially skin disease as listed in the S-chapter of the ICPC [see Additional file 2], within 14 days prior to their admission than controls (GP controls and hospital controls). We calculated odds ratios for the presence of GP consultations for all diseases, skin diseases and other diseases than skin diseases (cases/controls) and 95% confidence intervals (CI) using a conditional logistic regression model. We performed the same analysis for skin diseases within 30 days prior to the hospital admission of the cases. We repeated the latter analysis in a more strictly defined group (N = 44) of cases suffering from sepsis or severe bacteraemia and their matched controls. These cases were explicitly defined as being admitted to hospital due to sepsis, meningitis, acute osteomyelitis, acute pyelonefritis, acute mastoiditis, infectious arthritis or pneumonia. A two-sided p-value less than 0.05 was considered significant in all tests.

## Results

### Study population

The total general practice population included 88,307 children aged 0–17 years. We found 101 cases that could be matched with 597 GP controls and 583 hospital controls. Table 1 shows the baseline characteristics of cases and both control groups. Cases were comparable to their controls regarding socio-demographic characteristics.

### GP consultations

Sixty eight cases (67%) consulted the GP 161 times within 14 days prior to their hospital admission; five cases (5%) consulted the GP for a skin disease. Among the GP controls 67 consultations were made by 53 (9%) children within 14 days prior to the admission of the case they were linked to; nine controls (1.5%) consulted the GP for a skin disease. In the same period 255 (43.7%) children among the hospital controls consulted their GP 477 times; of these children 20 (3.4%) presented a skin disease. Table 2 shows which skin diseases were presented to the GP by cases and controls.

**Table 2:** GP consultation for skin diseases within 14 days prior to hospital admission of cases

Diagnoses	ICPC^1^	Cases (N = 101)	GP Controls^2 ^(N = 597)	Hospital Controls^3 ^(N = 583)
Pruritis	**S02**	0	1	0
Rash localized	**S06**	0	0	1
Skin infection post-traumatic	**S11**	0	0	1
Insect bite/sting	**S12**	0	1	0
Burn/scald	**S14**	0	3	1
Bruise/contusion	**S16**	0	0	1
Laceration/cut	**S18**	0	0	1
Dermatophytosis	**S74**	1	0	1
Moniliasis/candidiasis skin	**S75**	1	2	4
Naevus/mole	**S82**	0	0	1
Impetigo	**S84**	0	1	2
Dermatitis seborrhoeic	**S86**	0	0	2
Dermatitis/atopic eczema	**S87**	2	2	4
Dermatitis contact/allergic	**S88**	0	0	2
Diaper rash	**S89**	0	0	2
Sebaceous cyst	**S93**	1	0	0
Molluscum contagiosum	**S95**	0	1	0
Urticaria	**S98**	0	0	1

1 = International Classification of Primary Care

2 = control group randomly selected from the general practitioners' (GP) patient lists irrespective of hospital admission and GP consultation

3 = control group randomly sampled from those children who were hospitalized for other reasons than sepsis or bacteraemia

Children who were primarily admitted to hospital for a skin disease (N = 29) and excluded from analysis had the following diagnosis at discharge: skin abscesses, cellulitis, erysipelas, impetigo, infected finger/toe, paronychia and local skin infections. Of these children 14 (48%) consulted the GP 28 times within 14 days prior to their hospital admission. Eight children (28%) consulted the GP for a skin disease.

### Strengths of relationships

Table 3A shows the odds ratios (cases/controls) for whether or not a GP was consulted stratified for skin diseases and other diseases than skin diseases within 14 days prior to the hospital admission of the cases for children aged 0–17 years. Compared to their controls, more cases consulted the GP. The odds ratio for skin diseases (cases/GP controls) was 3.4 (95% CI: [1.1–10.8], p = 0.03) and 1.4 (95% CI: [0.5–3.9], p = 0.44) for cases versus hospital controls.

**Table 3:** A: GP consultations of children aged 0–17 years admitted for bacterial infections and matched controls: odds ratios, 95% confidence intervals and p-values B: GP consultations of children < 3 months admitted for bacterial infections and matched controls: odds ratios, 95% confidence intervals and p-values C: GP consultations of children aged 3 months to 17 years admitted for bacterial infections and matched controls: odds ratios, 95% confidence intervals and p-values

**(A)**		
Diagnoses according to ICPC^1^	Cases (N = 101) vs GP controls (N = 597)	Cases (N = 101) vs Hospital controls (N = 583)

**Skin diseases (S01 – S99)**	OR^2 ^3.4 [1.1–10.8], p = 0.03	OR 1.4 [0.5–3.9], p = 0.44
**Other diseases**	OR 33.0 [16.4–66.7], p < 0.0001	OR 2.8 [1.8–4.5], p < 0.0001
**All diseases**	OR 25.9 [13.6–49.4], p < 0.0001	OR 2.7 [1.7–4.2], p < 0.0001

**(B)**		

Diagnoses according to ICPC^1^	Cases (N = 9) vs GP controls (N = 46)	Cases (N = 9) vs Hospital controls (N = 54)

**Skin diseases (S01 – S99)**	OR^2 ^9.2 [0.81–106.1], p = 0.07	OR 4.0 [0.67–23.9], p = 0.12
**Other diseases**	OR 19.2 [2.2–164.0], p = 0.007	OR 5.8 [1.13–30.3], p = 0.03
**All diseases**	OR 15.3 [1.8–130.1], p = 0.012	OR 5.9 [1.13–30.3], p = 0.03

**(C)**		

Diagnoses according to ICPC^1^	Cases (N = 92) vs GP controls (N = 551)	Cases (N = 92) vs Hospital controls (N = 529)

**Skin diseases (S01 – S99)**	OR^2 ^2.5 [0.7–9.9], p = 0.17	OR 1.0 [0.3–3.5], p = 0.98
**Other diseases**	OR 34.8 [16.6–73.2], p < 0.0001	OR 2.6 [1.6–4.2], p < 0.0001
**All diseases**	OR 27.2 [13.7–53.2], p < 0.0001	OR 2.4 [1.5–4.0], p = 0.002

1 = International Classification of Primary Care

2 = Odds ratio

Table 3B and 3C show the odds ratios of skin diseases and other diseases for children younger than three months and for children aged three months to17 years respectively. Cases younger than three months showed an odds ratio (cases/GP controls) of 9.2 (95% CI: [08.1–106.1], p = 0.07). In this age group the odds ratio (cases/hospital controls) was 4.0 (95% CI: [0.67–23.9], p = 0.12). In all age groups significantly more cases consulted the GP for other diseases than skin diseases 14 days prior to their hospital admission compared to matched controls.

Repeated analysis of consultations for skin diseases within 30 days prior to the hospital admission of the cases showed similar results, as did repetition of the analysis restricted to the most severe cases (N = 44) and their controls.

## Discussion

We tested the null hypothesis that there is no difference between children admitted for sepsis or bacteraemia and other children as to consulting a GP for skin diseases in a period of 14 days before admission to hospital. We found that there is an association between skin diseases presented to the GP and subsequent hospitalization for sepsis or bacteraemia among GP controls but not for hospital controls.

We performed the same analysis in cases and controls younger than three months and found an even stronger relationship, though not significant. This lack of significance is probably due to the small number of cases in this age group.

From a clinical point of view the difference between cases and controls may not be very relevant. The probability that a case consulted the GP for skin diseases prior to their hospital admission is only about 5% and therefore not a point of departure for GPs to reduce the risk of sepsis and/or bacteraemia considerably by diagnosing and treating skin diseases appropriately. However, considering cases younger than 3 months (N = 9) about 22% consulted the GP for skin diseases prior to their hospital admission which means that GPs may have possibilities in this age group to reduce the risk of sepsis and/or bacteraemia considerably by diagnosing and treating skin diseases appropriately. We recommend replication of our study in a larger dataset for this age group.

Compared with both control groups our cases visited the GP about two times as high with both infectious skin diseases and atopic skin diseases as well, which could support the association between sepsis or bacteremia and infectious and atopic skin diseases [1,2,9,12,14].

In all age groups we found odds ratios concerning GP consultations for other diseases than skin diseases that are considerably high and significantly different (p < 0.0001) compared to the odds ratios for skin diseases. This finding indicates that there is a very strong association between GP consultations for other diseases than skin diseases, 14 days prior to hospital admission, and being hospitalized for sepsis or bacteraemia.

These two large and representative datasets enabled us to assess accurately odds ratios among cases and their matched controls and to test our hypothesis. By matching our cases and controls on age, gender and region we adjusted for differences concerning these variables and also for other socio-demographic characteristics (table 1). To limit the seasonal variation of the GP consultations we selected only the consultations that took place within 14 days prior to the admission date of the case to whom the controls were linked to.

Overall the odds ratio for a GP consultation concerning skin diseases among cases versus GP controls 14 days prior to the admission of the cases is higher compared to the odds ratio among cases versus hospital controls. Our findings are in accordance with an earlier finding by Infante-Rivard [22] that inferences of severe childhood diseases using hospital controls in comparison with population controls resulted in odds ratios closer to the null value.

## Conclusion

There is evidence that children who were admitted due to sepsis or bacteraemia consulted the GP more often for skin diseases prior to their admission, than other children, but the differences are not clinically relevant which means that there is little opportunity for GPs to reduce the risk of sepsis and/or bacteraemia considerably by diagnosing and treating skin diseases appropriately.

## Competing interests

The author(s) declare that they have no competing interests.

## Authors' contributions

RSAM and JCvdW designed the study. RSAM and SPW carried out the analyses, RSAM drafted the paper. All authors commented on draft versions and approved the final manuscript.

## Pre-publication history

The pre-publication history for this paper can be accessed here:



## Supplementary Material

Additional File 1 ICD-9 codes used for selection of sepsis and bacteraemia cases. discharge diagnoses related to sepsis or bacteraemia according to ICD-9 classification, used for selecting cases. Click here for file

Additional File 2 Chapter S (skin diseases) of the International Classification of Primary Care (ICPC). tabulation of all codes in chapter S (skin diseases) of the International Classification of Primary Care (ICPC). Click here for file
