# Supplementary material for: Association between skin diseases and severe bacterial infections in children: case-control study
Source: BMC Fam Pract. 2006 Aug 31;7:52. doi: 10.1186/1471-2296-7-52 (PMC1564399; doi:10.1186/1471-2296-7-52)
Supplement: Additional File 1 — ICD-9 codes used for selection of sepsis and bacteraemia cases. discharge diagnoses related to sepsis or bacteraemia according to ICD-9 classification, used for selecting cases. [file 1471-2296-7-52-S1.doc]

**ICD-9 codes used for selection of sepsis and bacteraemia cases**

To select our cases of sepsis and bacteraemia we used the ICD-9 classification.

We selected admissions with the following discharge codes and subcodes:

036 Meningococcal infection

036.0 Meningococcal meningitis

036.1 Meningococcal encephalitis

036.2 Acute meningococcaemie (meningococcal septicemia)

036.3 Waterhouse-Friderichsen

036.4 Meningococcal heart disease (carditis, endocarditis, myocarditis, pericarditis)

036.8 Other meningococcal infections (optic neuritis)

036.9 Meningococcal infection, unspecified

038 Septicaemia

038.0 Streptococcal septicaemia

038.1 Staphylococcal septicaemia

038.2 Pneumococcal septicaemia

038.3 Septicaemia due to anaerobes

038.4 Septicaemia due to other Gram-negative organisms (Haemophilus Influenzae, E.Coli, Pseudomonas, Serratia)

038.8 Other specified septicaemias

320 Bacterial meningitis, not elsewhere classified

320.0 Haemophilus meningitis

320.1 Pneumococcal meningitis

320.2 Streptococcal meningitis

320.3 Staphylococcal meningitis

320.8 Meningitis due to other specified bacteria

320.9 Meningitis due to unspecified bacterium

383 Mastoiditis and related conditions

383.0 Acute mastoiditis (abscess of mastoid, empyema of mastoid)

420 Acute pericarditis

421 Acute and subacute endocarditis

421.0 Acute and subacute bacterial endocarditis

481 Pneumococcal pneumonia

482 Other bacterial pneumonia

482.0 Pneumonia due to Klebsiella pneumoniae

482.1 Pneumonia due to Pseudomonas

482.2 Pneumonia due to H. Influenzae

482.3 Pneumonia due to Streptococcus

482.4 Pneumonia due to Staphylococcus

482.8 Pneumonia due to other specified bacteria

482.9 Bacterial pneumonia, unspecified

483 Pneumonia due to other speicified organism

513 Abscess of lung and mediastinum

513.0 Abscess of lung

- 1. Abscess of mediastinum

580 Acute glomerulonephritis (Includes: acute nephritis)

590 Infections of kidney

590.1 Acute pyelonephritis (acute pyelitis, acute pyonephrosis)

730 Osteomyelitis, periostitis, and other infections involving bone

730.0 Acute osteomyelitis

041 Bacterial infection in conditions classified elsewhere

041.0 Streptococcus

041.1 Staphylococcus

041.2 Pneumococcus

041.3 Friedlander’s bacillus

041.4 Escherichia coli (E.coli)

041.5 Haemophilus influenzae (H. influenzae)

041.6 Proteus (mirabilis, morganii)

041.7 Pseudomonas

041.8 Other

041.9 Bacterial infection, unspecified

711 Arthropathy associated with infections

711.0 Pyogenic arthritis (arthritis due to E. coli, H. influenzae, Pneumococ, Staphylococ, Streptococ)
