# Supplementary material for: Association between skin diseases and severe bacterial infections in children: case-control study
Source: BMC Fam Pract. 2006 Aug 31;7:52. doi: 10.1186/1471-2296-7-52 (PMC1564399; doi:10.1186/1471-2296-7-52)
Supplement: Additional File 2 — Chapter S (skin diseases) of the International Classification of Primary Care (ICPC). tabulation of all codes in chapter S (skin diseases) of the International Classification of Primary Care (ICPC). [file 1471-2296-7-52-S2.doc]

**Chapter S (skin diseases) of the International Classification of Primary Care (ICPC)**[19]:

S01 Pain / tenderness of skin

S02 Pruritus

S03 Warts

S04 Lump / swelling localized

S05 Lumps / swellings generalized

S06 Rash localized

S07 Rash generalized

S08 Skin colour change

S09 Infected finger / toe

S10 Boil / carbuncle

S11 Skin infection post-traumatic

S12 Insect bite / sting

S13 Animal / human bite

S14 Burn / scald

S15 Foreign body in skin

S16 Bruise / contusion

S17 Abrasion / scratch / blister

S18 Laceration / cut

S19 Skin injury other

S20 Corn / callosity

S21 Skin texture symptom / complaint

S22 Nail symptom / complaint

S23 Hair loss / baldness

S24 Hair / scalp symptom / complaint

S26 Fear of cancer of skin

S27 Fear of skin disease other

S28 Limited function / disability(s)

S29 Skin symptom / complaint other

S70 Herpes zoster

S71 Herpes simplex

S72 Scabies / other acariasis

S73 Pediculosis / skin infestation other

S74 Dermatophytosis

S75 Moniliasis / candidiasis skin

S76 Skin infection other

S77 Malignant neoplasm of skin

S78 Lipoma

S79 Neoplasm skin benign / unspecified

S80 Solar keratosis / sunburn

S81 Haemangioma / lymphangioma

S82 Naevus / mole

S83 Congenital skin anomaly other

S84 Impetigo

S85 Pilonidal cyst / fistula

S86 Dermatitis seborrhoeic

S87 Dermatitis / atopic eczema

S88 Dermatitis contact / allergic

S89 Diaper rash

S90 Pityriasis rosea

S91 Psoriasis

S92 Sweat gland disease

S93 Sebaceous cyst

S94 Ingrowing nail

S95 Molluscum contagiosum

S96 Acne

S97 Chronic ulcer skin

S98 Urticaria

S99 Skin disease other
